# Primary and secondary agonists can use P2X_1_ receptors as a major pathway to increase intracellular Ca^2+^ in the human platelet

**DOI:** 10.1111/j.1538-7836.2007.02525.x

**Published:** 2007-05-01

**Authors:** C Y E FUNG, C CENDANA, R W FARNDALE, M P MAHAUT-SMITH

**Affiliations:** * Department of Physiology, Development and Neuroscience, University of Cambridge Cambridge, UK; † Department of Biochemistry, University of Cambridge Cambridge, UK; ‡ Department of Cell Physiology and Pharmacology, University of Leicester Leicester, UK

**Keywords:** ATP, Ca^2+^, collagen, P2X_1_, thrombin, thromboxane A_2_

## Abstract

*See also* Nurden AT. Does ATP act through P2X_1_ receptors to regulate platelet activation and thrombus formation? This issue, pp 907–9.

In the platelet, it is well established that many G-protein- and tyrosine kinase-coupled receptors stimulate phospholipase-C-dependent Ca^2+^ mobilization; however, the extent to which secondary activation of adenosine 5′-triphosphate (ATP)-gated P2X_1_ receptors contributes to intracellular Ca^2+^ responses remains unclear. We now show that selective inhibition of P2X_1_ receptors substantially reduces the [Ca^2+^]_i_ increase evoked by several important agonists in human platelets; for collagen, thromboxane A_2_, thrombin, and adenosine 5′-diphoshate (ADP) the maximal effect was a reduction to 18%, 34%, 52%, and 69% of control, respectively. The direct contribution of P2X_1_ to the secondary Ca^2+^ response was far greater than that of either P2Y receptors activated by co-released ADP, or via synergistic P2X_1_:P2Y interactions. The relative contribution of P2X_1_ to the peak Ca^2+^ increase varied with the strength of the initial stimulus, being greater at low compared to high levels of stimulation for both glycoprotein VI and PAR-1, whereas P2X_1_ contributed equally at both low and high levels of stimulation of thromboxane A_2_ receptors. In contrast, only strong stimulation of P2Y receptors resulted in significant P2X_1_ receptor activation. ATP release was detected by soluble luciferin:luciferase in response to all agonists that stimulated secondary P2X_1_ receptor activation. However, P2X_1_ receptors were stimulated earlier and to a greater extent than predicted from the average ATP release, which can be accounted for by a predominantly autocrine mechanism of activation. Given the central role of [Ca^2+^]_i_ increases in platelet activation, these studies indicate that ATP should be considered alongside ADP and thromboxane A_2_ as a significant secondary platelet agonist.

## Introduction

Platelets express three receptors gated by extracellular nucleotides: P2X_1_, P2Y_1_, and P2Y_12_ [1,2]. P2X receptors are Ca^2+^-permeable ligand-gated non-selective cation channels, whereas P2Y receptors are seven transmembrane domain receptors that couple to cellular responses via the activation of heterotrimeric G-proteins [3]. Although adenosine 5′-diphoshate (ADP) was initially believed to act at all three human platelet P2 receptors, it is now clear that ADP and adenosine 5′-triphosphate (ATP) are selective physiological agonists at the platelet P2Y and P2X_1_ receptors, respectively [4]. The important role that P2Y_1_ and P2Y_12_ receptors play during hemostasis and thrombosis is well established [5,6]. In contrast, the relevance of P2X_1_ receptors to platelet function has been questioned because they rapidly desensitize [4] and their selective activation *in vitro* evokes a transient shape change without significant aggregation [7]. However, murine models demonstrate an important contribution of this ATP-gated non-selective cation channel to thrombosis, particularly in small arteries [8,9]. One explanation for the major contribution of P2X_1_ to platelet activation *in vivo* is that ATP released from dense granules contributes to signaling events following initial stimulation by other agonists. In support of this, human and murine studies have shown a role for P2X_1_ in the aggregation responses to low doses of collagen and thrombin [8,10,11]. Whilst much work has been conducted to characterize P2X_1_ involvement in downstream platelet function, the extent to which P2X_1_ acts independently or in synergy with other secondary mediators during the early stages of platelet activation remains unclear. In the present study, we have used measurements of the key platelet second messenger, intracellular Ca^2+^ ([Ca^2+^]_i_), to examine the relative importance of P2X_1_ receptors alone and in combination with P2Y receptors in the initial responses to a number of major agonists.

## Methods

### Preparation of platelet suspensions

Fura-2-loaded washed suspensions of human platelets from informed, consenting donors were prepared using acid citrate dextrose anticoagulant and treated with aspirin (100 μm) and type VII apyrase (0.32 U mL^−1^) as described elsewhere [7]. The study was approved by the University of Cambridge Human Biology Research Ethics Committee. Platelets were resuspended in nominally Ca^2+^-free saline (in mm: 145 NaCl, 5 KCl, 1 MgCl_2_, 10 HEPES, 10 glucose, titrated to pH 7.35 with NaOH) with type VII apyrase (0.32 U mL^−1^). 2 mm CaCl_2_ or 2 mm MgCl_2_ was added to the cuvette 30 s prior to the agonist for studies in the presence and absence of external Ca^2+^, respectively.

### [Ca^2+^]_i_ measurements

Fura-2 ratiometric fluorescence measurements were conducted at 37 °C in a Cairn spectrofluorimeter system (Cairn Research Limited, Faversham, Kent, UK) and converted to [Ca^2+^]_i_ as described elsewhere using a dissociation constant for Ca^2+^ of 224 nm [7].

### Reagents

Collagen type I, as a suspension of native fibers from bovine tendon, was the gift of Ethicon Corporation (Somerville, NJ, USA). Collagen-related peptide (CRP) with the sequence H–GPC–(GPO)_10_–GPCG–NH_2_ was prepared and cross-linked as described by Morton *et al.* [12]. ADP was treated with hexokinase as described previously [13] and ATP levels assessed by bioluminescent measurements (ATP Assay Kit, Calbiochem-Novabiochem UK Ltd, Nottingham, UK, or Chromo-lume Kit, Labmedics, Manchester, UK) using a Model 400 lumi-aggregometer (Chrono-log Corporation, Havertown, PA, USA). U46619 and thapsigargin were from Calbiochem-Novabiochem UK Ltd. Cangrelor (AR-C69931MX) was a kind donation from AstraZeneca (Moindal, Sweden). All other reagents, including thrombin receptor activating peptide (TRAP) specific for PAR1, ADP, α,β-meATP, NF449 and MRS2179 were from Sigma-Aldrich (Poole, UK).

### Luminescence measurement of ATP secretion

ATP secretion from washed platelet suspensions was measured in a Model 400 lumi-aggregometer as above, following the kit manufacturer’s guidelines. The luminescence channel output was amplified tenfold before acquisition to computer. Platelets used in luminescence studies were loaded with fura-2 to provide parallel measurements of ATP secretion and [Ca^2+^]_i_ under closely matched conditions. 600 nmα,β-meATP did not interfere with the luciferin-luciferase assay for ATP (Chen, Fung, and Mahaut-Smith, unpublished observations).

### Analysis

Sample records of [Ca^2+^]_i_ are representative of experiments from ≥ four separate donors. The magnitude of Ca^2+^ responses was assessed from the peak increase above prestimulus level. Average values represent the mean ± SEM, with statistical analysis performed using Student’s paired *t*-test. Significance is indicated at levels of 0.05 (*), 0.01 (**), 0.005 (***), and 0.001 (****).

## Results

### Conditions for maximal, selective inhibition of individual platelet P2 receptor subtypes

**Fig. 1:**
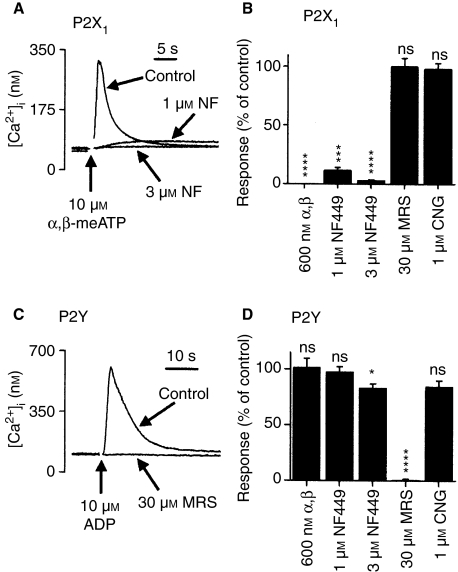
Conditions for maximal, selective inhibition of P2X_1_ receptors. Effect of different P2 receptor antagonists or pre-addition of α,β-meATP on [Ca^2^]_i_ responses via P2X_1_ receptors (A,B, 10 μmα,β-meATP in saline with 2 mm Ca^2+^) or P2Y receptors (C,D, hexokinase-treated ADP in nominally Ca^2+^-free saline; 10 μm ADP for MRS 2179, 1 μm ADP for other compounds). (A), (C) Representative recordings. (B), (D) Average peak responses, as percentage of paired controls. 600 nmα,β-meATP was added 90 s prior to the test agonist. In all figures, the asterisks above each bar indicate the ‘*P*-value’ relative to the paired control; ns, not significant; α,β, α,β-meATP; MRS, MRS 2179; CNG, cangrelor.

To assess the relative contribution of the three platelet P2 receptors to [Ca^2+^]_i_ responses, we first derived conditions that provide maximal block, but with strict limits on selectivity. Thus, whilst 3 μm was the minimum concentration of the suramin derivative NF449 [14] required to completely block P2X_1_ receptor-evoked Ca^2+^ influx, it also significantly reduced the ADP (1 μm)-evoked Ca^2+^ release via P2Y receptors to 83 ± 4% of control (Fig. 1). However, 1 μm NF449 had no significant effect on ADP-evoked Ca^2+^ release, yet still reduced the peak response via P2X_1_ receptors to 11 ± 2% of control (Fig. 1B,D). In comparison, P2Y responses to 10 μm ADP were reduced to negligible levels (0.3 ± 0.2%) by 30 μm MRS2179 (Fig. 1C,D), a concentration which has no effect at P2Y_12_ receptors [15] or P2X_1_ responses (Fig. 1B). Higher concentrations of MRS2179 were not employed as they started to inhibit P2X_1_ receptors (Fung, unpublished observations). However, 30 μm MRS2179 can be considered as an effective blocker of most, if not all secondary Ca^2+^ responses via P2Y receptors as maximal local extracellular concentrations of ADP are likely to be in the range 10–20 μm. This estimation is based upon the surface-attached luciferase measurements of ATP by Beigi *et al.* [16], and the fact that ATP and ADP are stored at equal concentrations in the dense granules of human platelets [17]. Because 1 μm cangrelor also had no significant effect on P2X_1_ (Fig. 1B), but maximally inhibits P2Y_12_ receptor responses [18] without affecting P2Y_1_ receptors [19], these results establish that 1 μm NF449, 1 μm cangrelor and 30 μm MRS2179 provide maximal, selective antagonism of P2X_1_, P2Y_12_ and P2Y_1_ receptors, respectively. An additional means to selectively inhibit P2X_1_ receptor Ca^2+^ influx is desensitization in Ca^2+^-free medium with α,β-meATP [10,20], which at 600 nm completely abolished P2X_1_ receptor-evoked Ca^2+^ increases without influencing ADP-evoked Ca^2+^ responses (Fig. 1B,D).

### The major role of P2X_1_ receptors in collagen-evoked Ca^2+^ increases does not require co-activation of P2Y receptors and principally involves glycoprotein VI

**Fig. 2:**
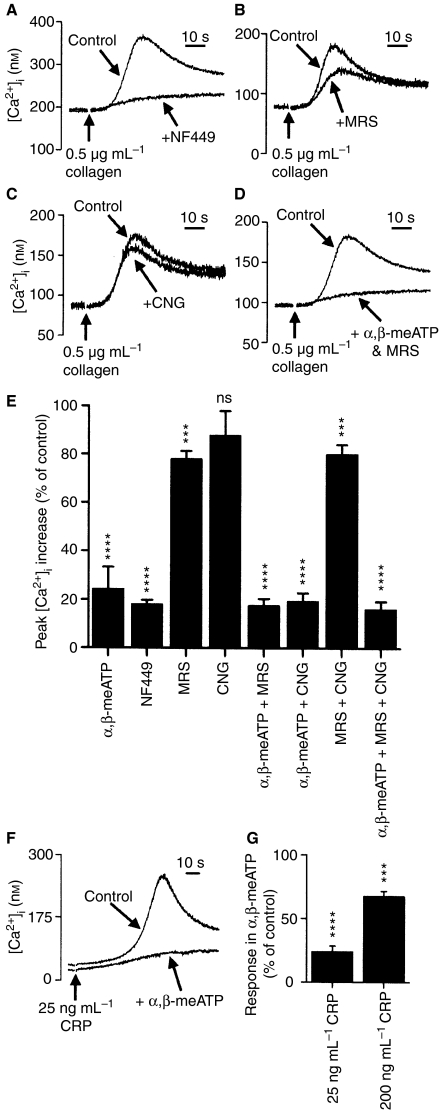
The major role of P2X_1_ in collagen-evoked Ca^2+^ signaling via glycoprotein (GP) VI does not rely upon co-activation of P2Y receptors. Representative (A–D, F) and average (E, G) [Ca^2+^]_i_ responses to collagen (0.5 μg mL^−1^, A–E) or collagen-related peptide (CRP; 25 or 250 ng mL^−1^; F, G). Average responses are shown as the percentage of a paired control. Responses were studied in the presence and absence of α,β-meATP (600 nm, added 90 s prior to agonist), NF449 (1 μm), MRS 2179 (MRS, 30 μm) and cangrelor (CNG, 1 μm), or a combination of these blockers as shown, all added 60 s before agonist. All experiments were conducted in the presence of external Ca^2+^ (2 mm).

Selective inhibition of P2X_1_ receptors with 1 μm NF449 caused a large reduction of the Ca^2+^ responses stimulated by a low dose of collagen (0.5 μg mL^−1^; Fig. 2A). The peak was reduced to 18 ± 2% (*P* < 0.05) of control, similar to the effect of P2X_1_ desensitization with 600 nmα,β-meATP (reduction to 25 ± 9% of control; Fig. 2E). Importantly, this confirms that α,β-meATP predesensitization has equivalent effects to simple direct blockade with NF449, and desensitization was used as a preferred tool as it permitted greater selective reduction of P2X_1_ receptor signals. The contribution of P2X_1_ receptors to collagen-evoked Ca^2+^ responses was far greater than that of P2Y receptors, as the response was reduced to only 78 ± 3%, 88 ± 10% and 80 ± 4%, respectively, for MRS2179, cangrelor and these two inhibitors combined. Combined blockade of P2X_1_ and either P2Y_1_ or both P2Y_1_ and P2Y_12_ receptors was not significantly different from block/desensitization of P2X_1_ alone (*P* > 0.05; Fig. 2E). Thus, we were unable to further explore possible synergy between P2X_1_ and P2Y receptors, as reported previously in the platelet [21]. However, this synergy may account for the lack of effect of MRS2179 on top of α,β-meATP despite a small but significant effect of MRS 2179 on its own.

Selective stimulation of glycoprotein (GP) VI with 25 ng mL^−1^ CRP [22] evoked a [Ca^2+^]_i_ response comparable to 0.5 μg mL^−1^ collagen, which was reduced to 24 ± 5% of control by α,β-meATP pre-addition (Fig. 2F), similar to the effect of P2X_1_ inhibition on low collagen concentrations. As observed for collagen [20], the percentage contribution of P2X_1_ to GPVI-dependent signals was less at higher concentrations of CRP (for example α,β-meATP reduced the response to 200 ng mL^−1^ CRP to only 68 ± 4% of control; Fig. 2G). Together these data suggest that GPVI represents the receptor by which collagen stimulates secondary activation of P2X_1_ receptors.

### P2X_1_ receptor inhibition by NF449, or desensitization by α,β-meATP, does not block other platelet Ca^2+^ entry pathways

**Fig. 3:**
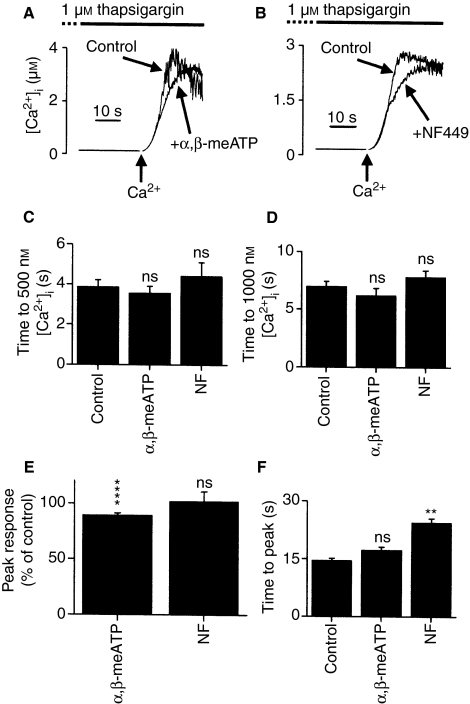
α,β-meATP and NF449 do not block store-dependent Ca^2+^ influx. Store-dependent Ca^2+^ influx was measured by addition of 2 mm Ca^2+^ after depletion of intracellular Ca^2+^ stores in nominally Ca^2+^-free saline with the endomembrane Ca^2+^-ATPase inhibitor, thapsigargin (1 μm). 5 min after thapsigargin treatment, 600 nmα,β-meATP, 1 μm NF449 or a water-vehicle control were added, followed a further 90 s later by 2 mm Ca^2+^. Representative traces are shown for α,β-meATP (A) and NF449 (B) with their paired control. The average rate of initial Ca^2+^ increase, as a direct measurement of store-dependent Ca^2+^ influx, was assessed as the time to 500 nm (C) or 1000 nm (D), while the overall response to Ca^2+^ re-addition was assessed from the peak increase (E) and time to peak (F).

Platelet G-protein-coupled or tyrosine kinase-linked receptors have been previously proposed to stimulate Ca^2+^ influx via store-dependent and store-independent pathways [23,24]. We have recently shown that NF449 and α,β-meATP do not affect platelet store-independent cation currents using direct electrophysiological measurements in the megakaryocyte [13]. To assess effects on store-dependent Ca^2+^ influx, intracellular Ca^2+^ stores were depleted for 5 min with 1 μm thapsigargin in Ca^2+^-free medium followed by addition of external Ca^2+^ (Fig. 3). Neither 600 nmα,β-meATP nor 1 μm NF449 had any significant effect on store-dependent Ca^2+^ influx as the time to reach [Ca^2+^]_i_ levels of 500 or 1000 nm following Ca^2+^ re-addition were unaffected (Fig. 3A–D). The lack of effect of NF449 or α,β-meATP on the initial thapsigargin-evoked Ca^2+^ influx also confirms that these P2X_1_-inhibiting reagents do not substantially alter the platelet membrane potential. However, the later phase of the Ca^2+^ increase in these re-addition experiments was slightly attenuated or delayed by P2X_1_ receptor inhibition, although this was only significant for the peak response for α,β-meATP pre-addition (decrease to 89 ± 2% of control; *P* < 0.001) and the time to peak for 1 μm NF449 (increase to 167 ± 18% of control; *P* < 0.05). A reasonable explanation for these effects is that high levels of Ca^2+^ lead to ATP secretion [25], which amplifies/accelerates the peak Ca^2+^ response through P2X_1_ receptor activation in parallel to store-mediated Ca^2+^ entry.

### Role of P2X_1_ receptors in thrombin-evoked Ca^2+^-responses

**Fig. 4:**
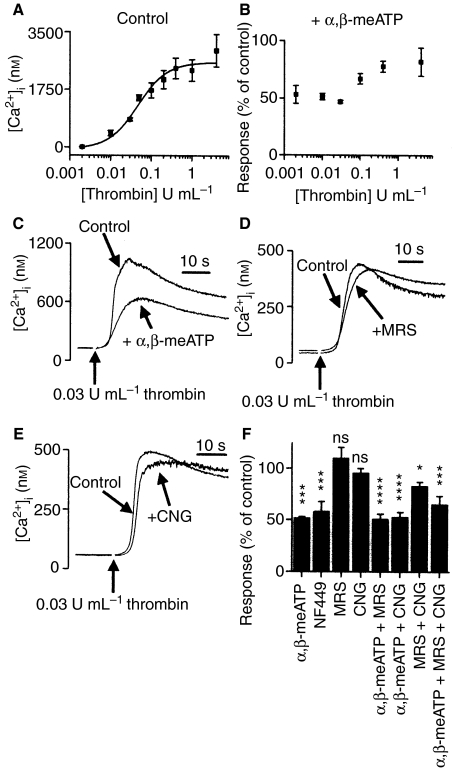
P2X_1_ contributes to thrombin-evoked Ca^2+^ responses, particularly at low–mid-range levels of PAR receptor stimulation. (A) Dose–response curve for the peak thrombin-evoked Ca^2+^ increase; the data were fit by a logistic curve with an EC_50_ of 0.032 ± 0.009 U mL^−1^ and a slope of 1.63 ± 0.75. (B) Effect of P2X_1_ receptor inhibition (90 s pre-exposure to 600 nmα,β-meATP) on the peak thrombin-evoked Ca^2+^ increase across a range of thrombin concentrations. (C)–(F) Representative (C–E) and average (F) responses (as a percentage of a paired control) to 0.03 U mL^−1^ thrombin following treatment with 600 nmα,β-meATP, 1.0 µm NF449, 30 µm MRS2179 (MRS) and 1.0 μm cangrelor (CNG), individually or in combination. All experiments were conducted in the presence of external Ca^2+^ (2 mm).

We next assessed the importance of P2X_1_ receptors during responses to thrombin, the most potent known Ca^2+^-mobilizing platelet agonist. The concentration–response curve for thrombin (0.001–4.0 U mL^−1^) in our platelet preparation is shown in Fig. 4A. Desensitization of P2X_1_ receptors reduced the peak response to thrombin in a concentration-dependent manner, which was maximal at low to mid-range thrombin concentrations (0.01–0.03 U mL^−1^; see concentration–response relationship in Fig. 4B and sample records at 0.03 U mL^−1^ in Fig. 4C). In the absence of external Ca^2+^, α,β-meATP had no effect on the peak [Ca^2+^]_i_ increase evoked by 0.03 U mL^−1^ thrombin (97 ± 2% of the control response; *P* > 0.05; data not shown), thus this nucleotide does not affect stored Ca^2+^ levels or thrombin receptors.

At a thrombin concentration close to the EC_50_ (0.03 U mL^−1^), no significant effect was observed for MRS2179 or cangrelor on the peak thrombin-evoked Ca^2+^ increase (96 ± 5% and 110 ± 10% of control, respectively; *P* > 0.05; Fig. 4D–F). This compares with reductions in peak Ca^2+^ responses to 52 ± 1% and 58 ± 10% of control with α,β-meATP and NF449, respectively. Furthermore, this contribution of P2X_1_ was not dependent upon interactions with P2Y receptors as α,β-meATP reduced thrombin-evoked Ca^2+^ increases to the same extent in the presence or absence of both MRS 2179 and cangrelor (*P* > 0.05; Fig. 4F). The receptor through which thrombin achieved P2X_1_ stimulation was mainly PAR-1 as the response to an EC_50_ concentration of TRAP specific for PAR-1 (10 μm) was reduced to 50 ± 6% of the control response by α,β-meATP (not shown), which is not significantly different to the effect of losing P2X_1_ receptor function during stimulation with 0.03 U mL^−1^ thrombin (*P* > 0.05).

### Role of P2X_1_ during activation by the secondary agonists ADP and thromboxane A_2_

**Fig. 5:**
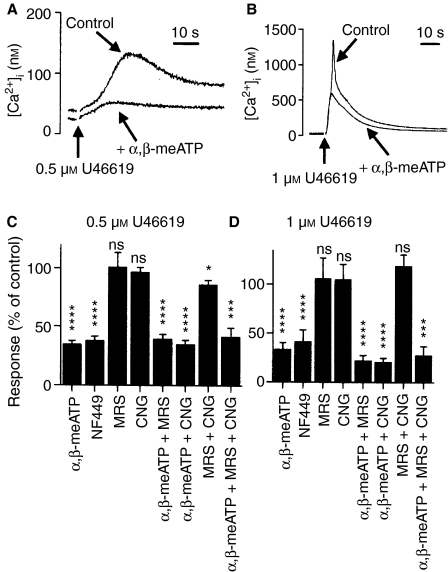
P2X_1_ receptors contribute to thromboxane A_2_ receptor-evoked Ca^2+^ increases at both high and low levels of stimulation. Representative (A,B) and average (C,D) [Ca^2+^]_i_ responses to the thromboxane A_2_ mimetic, U46619, at concentrations that evoke small (0.5 μm; A,C) and large (1 μm; B,D) peak [Ca^2+^]_i_ responses. (A) and (B) show representative paired traces with and without 600 nmα,β-meATP (90 s exposure) to desensitize P2X_1_ receptors. (C) and (D) show average peak [Ca^2+^]_i_ increases as a percentage of a paired control following treatment with 600 nmα,β-meATP, 1.0 µm NF449, 30 μm MRS2179 (MRS), 1.0 µm cangrelor (CNG), individually or in combination. All experiments were conducted in the presence of external Ca^2+^ (2 mm).

We also examined the role of P2X_1_ receptors in response to direct stimulation by thromboxane A_2_ and ADP, two further platelet agonists with important roles in hemostasis and thrombosis. The stable thromboxane A_2_ analogue, U46619, activated its full range of [Ca^2+^]_i_ increases over only a narrow range of concentrations; consequently, small and large responses could be achieved with 0.5 and 1.0 μm U46619, respectively. Inhibition of P2X_1_ receptors caused a marked decrease in the peak [Ca^2+^]_i_ increase at both these concentrations; peak responses to 1 and 0.5 μm U46619 were reduced to 42 ± 11% and 38 ± 4%, respectively, by 1 μm NF449 and 34 ± 7% and 35 ± 3%, respectively, after α,β-meATP (Fig. 5C,D). Block of P2Y receptors had only small effects on [Ca^2+^]_i_ responses to U46619 and, as observed for collagen and thrombin, block of both P2X_1_ and P2Y receptors was not significantly different (*P* > 0.05) to the effect of P2X_1_ desensitization alone.

**Fig. 6:**
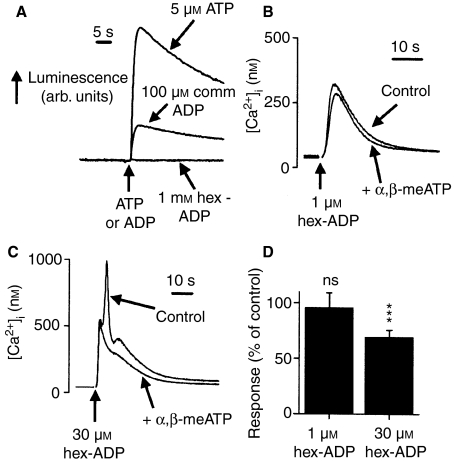
P2X_1_ receptors contribute to P2Y receptor-evoked Ca^2+^ responses only at high levels of stimulation. (A) ATP-dependent luminescence signal (from a luciferin:luciferase assay, in arbitrary units) following addition of 5 μm ATP, 100 μm commercial ADP (comm. ADP) and 1 mm ADP after treatment with hexokinase (hex-ADP). (B,C) Representative paired [Ca^2+^]_i_ responses to 1 or 30 μm ADP (hexokinase-treated) in the presence or absence of 600 nmα,β-meATP (90 s, to desensitize P2X_1_ receptors). (D) Effect of P2X_1_ desensitization on peak ADP-evoked [Ca^2+^]_i_ responses as a percentage of a paired control. All experiments were conducted in the presence of external Ca^2+^ (2 mm).

The role of P2X_1_ receptors in ADP-evoked responses is difficult to study because of contamination of commercial samples with ATP [26]; however, hexokinase treatment reduces the level of ATP to negligible levels (Fig. 6A). Desensitization of P2X_1_ receptors had no effect on the Ca^2+^ response to low concentrations of ATP-free ADP (1 μm, *P* > 0.05; Fig. 6B,D). However, maximal stimulation of P2Y receptors with 30 μm hexokinase-treated ADP generated a noticeable secondary phase of [Ca^2+^]_i_ increase, which was abolished by α,β-meATP pre-addition (Fig. 6C). The secondary Ca^2+^ spike was variable between donors, and overall the average peak ADP-evoked Ca^2+^ increase was reduced to 69 ± 7% (*P* < 0.01). The secondary Ca^2+^ spike was not a result of direct activation of P2X_1_ receptors by contaminating ATP for two reasons. First, we estimate that the concentration of ATP in 30 μm hexokinase-treated ADP is less than 1 nm, a level that is unable to activate P2X_1_ receptors. Secondly, any direct P2X_1_ receptor activation would be rapid; yet the amplitude and rate of initial ADP-evoked Ca^2+^ increase was not altered by α,β-meATP pre-addition (Fig. 6C). Therefore, it is likely that 30 μm ADP stimulates P2X_1_ via release of ATP (see below).

### Early dense granule secretion generates only nanomolar levels of bulk phase ATP

**Fig. 7:**
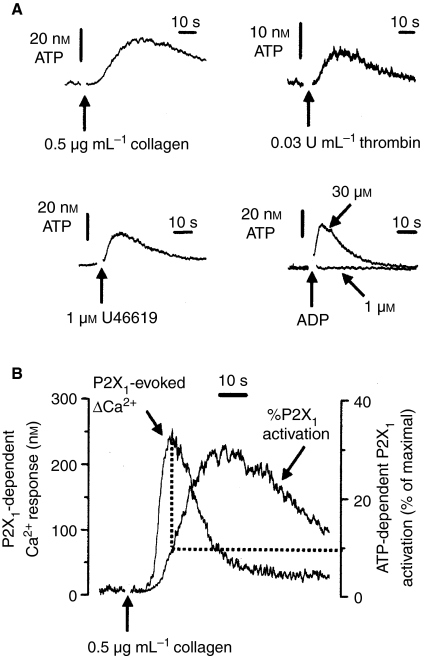
Comparison of average bulk phase ATP levels and intracellular Ca^2+^ responses following stimulation by multiple agonists; evidence for autocrine activation of P2X_1_ receptors. (A) Effect of collagen (0.5 μg mL^−1^), thrombin (0.03 U mL^−1^), U46619 (1 μm) and ADP (1 and 30 μm) on extracellular ATP levels measured using the luciferin–luciferase firefly assay. The peak luminescence signal for a range of known ATP concentrations was used to calibrate the agonist-evoked signals. (B) Superimposition of the P2X_1_-dependent Ca^2+^ increase and ATP secretion evoked by 0.5 μg mL^−1^ collagen. The dashed line corresponds to the peak contribution by P2X_1_, which occurs when the average extracellular ATP level is predicted to stimulate 10% of maximal P2X_1_ receptor activation. All experiments were conducted in the presence of external Ca^2+^ (2 mm).

To examine the relationship between ATP release and the time course of secondary P2X_1_ receptor activation, total ATP levels were measured using luciferin/luciferase added to washed platelet suspensions. Early ATP release was detected in response to 30 μm ADP but not in response to 1 μm ADP (Fig. 7A), which together with the data in Fig. 6 is consistent with a requirement for ATP release for P2X_1_ activation. Furthermore, early ATP release was observed in response to collagen, thrombin and U46619 at concentrations of these agonists that induce substantial P2X_1_ receptor activation (Fig. 7A). However, in all cases the early peak ATP increase was only 10–30 nm during the first 30 s, when P2X_1_ receptors played a major role in the [Ca^2+^]_i_ increases. To examine the temporal relationship between secretion and Ca^2+^-mobilization, the P2X_1_-dependent Ca^2+^ response to 0.5 μg mL^−1^ collagen was derived from recordings in the presence and absence of α,β-meATP and compared to the percentage P2X_1_ receptor activation. The latter was calculated from the ATP concentration–response relationship of P2X_1_ receptors following complete inhibition of G-protein-coupled receptor Ca^2+^ responses with a prostacyclin concentration that has no effect on P2X_1_ receptors (Fung and Mahaut-Smith, unpublished observations; Fig. 7B). This temporal comparison shows that the average extracellular ATP concentration increases with a delayed time course relative to the P2X_1_-dependent Ca^2+^ increase. This difference can be accounted for by a predominantly autocrine mechanism of P2X_1_ receptor activation as ATP increases near the sites of granule release will be larger and more rapid than in the bulk phase.

## Discussion

An increase in [Ca^2+^]_i_ is used by a wide range of platelet agonists as a key signal to activate downstream events. On its own, [Ca^2+^]_i_ increases within the physiological range can activate most functional responses in the platelet including shape change, aggregation, secretion and procoagulant activity, although Ca^2+^-mobilization normally operates in tandem with other signaling pathways such as tyrosine kinases, PI 3-kinase and inhibition of cAMP production [25,27]. The central role of Ca^2+^ in hemostasis and thrombosis is demonstrated by the ability of cytosolic BAPTA (a Ca^2+^ chelator) to block or markedly inhibit a number of mainstream platelet responses such as inside-out activation of α_IIb_β_3_, procoagulant activity and arachidonate production [28–30]. The present study shows, for the first time, that secondary activation of P2X_1_ receptors represents a major means by which both G-protein-coupled and tyrosine kinase-coupled receptors elevate [Ca^2+^]_i_ independently of ADP-activated P2Y receptors. This enhancement of the initial Ca^2+^ responses may explain the previously reported ability of P2X_1_ receptors to potentiate aggregation at low levels of collagen and thrombin [8,10,11]. This general role of P2X_1_ receptors as a secondary Ca^2+^ influx pathway could also help explain the resistance to thrombosis displayed by P2X_1_^−/−^ mice [8] and the enhanced thrombotic phenotype of mice overexpressing human P2X_1_ receptors [9].

Platelet receptors coupled to phospholipase-C activation mobilize Ca^2+^ by a combination of IP_3_-dependent store release and influx across the plasma membrane [25]. The current view is that these influx pathways consist of ion channels activated by either intracellular Ca^2+^ store depletion (store-operated Ca^2+^ influx) or the products of PLC such as diacylglycerol (termed store-independent Ca^2+^ influx) [13,23,24]. We now show that P2X_1_ receptors should be considered as a significant additional contributor to early Ca^2+^ influx in the platelet following stimulation by agonists acting via G-protein-coupled receptors and tyrosine-kinase-coupled receptors.

We have previously shown that P2X_1_ is able to potentiate the non-selective cation channel coupled to P2Y receptors in murine megakaryocytes and to accelerate and amplify the P2Y-evoked Ca^2+^ mobilization in suspensions of human platelets [21]. Although such synergy may exist when both P2X_1_ and P2Y receptors are active, the present study shows that P2X_1_ does not rely upon interactions with co-activated P2Y receptors to contribute as a secondary Ca^2+^-elevating pathway in the platelet. Serotonin release from dense granules also has little or no role in the Ca^2+^ responses in these experiments as a maximal concentration of serotonin (1 μm) evoked a Ca^2+^ increase of < 15 nm, and 100 nm methiothepin (a concentration that blocked responses to 1 μm serotonin) had no significant effect on the Ca^2+^ increase evoked by 1 μm U46619 (109.5 ± 13.2% of control; *P* > 0.05). A likely explanation for the important independent role of P2X_1_ is that the primary agonist already stimulates the phospholipase-C-dependent Ca^2+^ fluxes subsequently targeted by released ADP via P2Y receptors, whereas P2X_1_ receptor-cation channels represent a separate route for elevating Ca^2+^. In addition, we show that P2X_1_ receptors can be activated only when small total amounts of ATP are released, thus allowing this pathway to contribute at low levels of dense granule secretion. Moreover, our experiments were conducted in the presence of apyrase, indicating the ability of ATP to activate P2X_1_ receptors even in the presence of significant levels of ectonucleotidase activity. The rapid kinetics of P2X_1_ activation by its ligand [4] most likely account for its efficient activation even in the presence of ectonucleotides. However, this role for ATP should not reduce the well-established importance of other released compounds such as ADP and thromboxane A_2_. In particular, because P2X_1_ causes elevation of only Ca^2+^ (and Na^+^), stimulation of complementary signaling pathways by P2Y_12_ receptors remains crucial for collagen and thromboxaneA_2_ receptor-evoked aggregation [1,2].

Two pieces of evidence suggest that secondary activation of P2X_1_ receptors occurs via an autocrine rather than a paracrine manner. First, the bulk phase ATP level when P2X_1_ maximally contributes is sufficient to activate less than 10% of P2X_1_ receptors (Fig. 7B). Secondly, P2X_1_ receptors contribute to the collagen-evoked Ca^2+^ increase earlier than the average extracellular ATP increases (Fig. 7B). These observations can be explained when it is considered that, following secretion, the extracellular ATP concentration at the plasma membrane surface will increase earlier, and be far greater in magnitude compared to the average level measured by soluble luciferin:luciferase. Localization of P2X_1_ receptors at sites of secretion may also explain how ATP release can so efficiently activate P2X_1_ receptors. Although there is no direct evidence for such localization at present, lipid rafts may play an important role as these microdomains in platelets contain both P2X_1_ receptors and SNARE proteins, and raft disruption leads to inhibition of P2X_1_ responses and exocytosis [31,32].

In conclusion, we show that P2X_1_ receptors can represent a significant pathway for early Ca^2+^-mobilization following activation of a variety of major receptors linked through both G-proteins and tyrosine kinases in the platelet. Thus, P2X_1_ receptors should be considered alongside store-operated and store-independent channels as an important route for Ca^2+^ influx, and therefore to be a more significant potential antithrombotic target than previously recognized.
